# Standardization of Coagulation Factor V Reference Intervals, Prothrombin Time, and Activated Partial Thromboplastin Time in Mice for Use in Factor V Deficiency Pathological Models

**DOI:** 10.3389/fvets.2022.846216

**Published:** 2022-03-28

**Authors:** Juan A. De Pablo-Moreno, Antonio Liras, Luis Revuelta

**Affiliations:** ^1^Department of Genetics, Physiology and Microbiology, School of Biology, Complutense University, Madrid, Spain; ^2^Department of Physiology, School of Veterinary Medicine, Complutense University of Madrid, Madrid, Spain

**Keywords:** factor V, prothrombin time, activated partial thromboplastin time, mouse, measurement standardization, transfusion, pathological models, factor V deficiency

## Abstract

Factor V together with activated factor X forms the prothrombinase complex, which transforms prothrombin into thrombin. The *Mus musculus* species is characterized by very high levels of this factor and short clotting times, which hinders accurate measurements. For that reason, a detailed characterization of such parameters is indispensable. A method was designed as part of this study to provide an accurate determination and standardization of factor V levels, prothrombin time and activated partial thromboplastin time in *Mus musculus*. Those parameters were evaluated in a sample of 66 healthy animals using a semi-automated coagulometer and human diagnostic reagents in an attempt to determine the most appropriate time of day for the extractions. A mouse-based protocol was designed, capable of making corrections to the samples at dilutions of 1:100 for factor V and at of 1:3 for prothrombin time. The goal was to smoothen the calibration curves, which often present with steep slopes and narrow measurement ranges between one calibration point and another. It was found that the most stable period for blood sample extraction was that comprised between the first 6 h of light. No clinical differences were observed between the sexes and reference intervals were established for factor V (95.80% ± 18.14; 25.21 s ± 1.34), prothrombin time (104.31% ± 14.52; 16.85 s ± 1.32) and activated partial thromboplastin time (32.86 s ± 3.01). The results obtained are applicable to human or veterinary biomedical research, to transfusional medicine or to pathological models for diseases such as factor V deficiency.

## Introduction

Mice have long been the preferred species for biomedical research aimed at generating human models for congenital conditions (1, 2), particularly congenital coagulopathies (3). Murine models have been designed for hemophilia A (factor VIII deficiency) (4) and for hemophilia B (factor IX deficiency) (5). Factor V (FV) deficient animal models have been created for mice as well as for zebra fish, but the individuals born at full term died a few days later due to breakthrough bleeds or because they were subjected to different procedures (6–8). Studies have confirmed that FV plays an important role in embryogenesis as FV deficiency may result in mid-pregnancy miscarriages and even in death both in mice and zebra fish. Although there are no veterinary medicine studies on congenital FV deficiency, a description has been published of FV deficiency acquired from anti-FV antibodies in dogs (9).

Hemostasis is the body's physiological response to an injury to the vasculature. The process consists in the formation of a clot to occlude the lesion, with the participation of the vascular endothelium, platelet activation and the coagulation cascade (10). The FV protein plays an important coagulant and anticoagulant role in the clotting process but must previously convert to activated FV (FVa). Together with activated factor X, factor V forms the prothrombinase complex, which transforms prothrombin into thrombin (10, 11). Human FV is 80% homologous with murine FV, with the B domain being the least conserved structure of the protein, as is also the case with other animal species and other coagulation factors (12, 13).

FV levels in mice are exceptionally high (14–16). Different FV reference levels have been proposed for mouse models based on the values established for human plasma (1,000 mU/mL). To that effect, standard calibration curves have been developed using photometric measurements (4,632 mU/mL ± 1,066) (13) or one-stage chronometric assays (2,920 mU/mL ± 89) (15).

Activated partial thromboplastin time (APTT) and prothrombin time (PT) are coagulometric parameters used to characterize the coagulation process (17). PT measures the tissue factor pathway by estimating the behavior of factors VII, V, X, prothrombin, fibrinogen and fibrin (17, 18). The PT reference value for mice is 9–12 s, although it is very much dependent on lineage (19). APTT estimates the performance of factors XII, XI, X, IX, VIII, V, prothrombin, fibrinogen and fibrin (17, 18). The APTT reference value in mice ranges from 20 to over 30 s, also depending on lineage (19).

*Mus musculus* is characterized by very high FV plasma levels, which hinders accurate measurements and makes a detailed characterization of this parameter indispensable. Characterization of PT and APTT is also required as FV is involved with both parameters, making it necessary to implement INR-based corrections. This study describes a method for accurately determining and standardizing coagulation FV levels as well as PT and APTT in *Mus musculus* on the basis of the values obtained from human plasma and from a plasma pool prepared with plasma samples of a population of healthy mice. The method presented is intended to contribute to establishing reference intervals and detecting inter-individual differences.

## Materials and Methods

This research project was carried out in compliance with the Spanish legislation as set out in Royal Decree 53/2013 and Directive 2010/63/EU. All procedures used were approved by the School of Veterinary Medicine of Madrid's Complutense University, the Ethics Committee on Animal Experimentation of the Complutense University, and the Madrid Regional Government (PROEX 258.1/21).

### Animals

Sixty-six healthy mice were used, 33 male and 33 female, donated by the MOSEVAR group. The mice were derived from inbred crosses with strains CBA, C57BL and BALB/c (20). All individuals were 8 months of age and had a mean weight of 39.35 ± 7.78 grams. Animals were kept under 12 h of light and 12 of dark, with water and food at libitum (Altromin International, Lage, Germany). Veterinarian control was enforced.

### Study Design

Individuals were divided into two sex-specific groups, containing 33 mice each. Calibration was performed using commercially available calibrators (Unicalibrator, Diagnostica Stago S.A.S. Barcelona, Spain) and calibration curves with plasma pools were prepared from six randomly selected mice (3 males and 3 females) at dark hour 0 (beginning of the dark period) and light hour 0 (beginning of the light period). A work schedule was created for carrying out a duplicate analysis of the plasma pools from six randomly selected individuals (3 males and 3 females) at every hour during the 24 h of the day. All animals recovered their normal volemic status before the next extraction. The hourly variations of the cohort's parameters were analyzed to determine the best extraction time for each individual. Individual measurements were made for all the individuals in the sample during the established period.

### Blood Collection

The extraction of blood to create the plasma pools was performed without anesthesia from the submandibular vein with a 23G needle. Individual measurements were made by extracting blood via intracardiac injection prior to which animals were anesthetized by intraperitoneal administration of a mixture of ketamine (Richer Pharma, Austria) and xylazine (Calier, Barcelona, Spain) [100 mg/kg // 10 mg/kg]. Following the intracardiac extraction, animals were euthanized by cervical dislocation. All samples were collected in tubes containing 0.5 mL sodium citrate buffer at 3.2% (0.109 M) (Vacutest Kima, Arzegrande, Padua, Italy); the blood/citrate ratio was 1:9. Samples were centrifuged at 2,500x*g* for 15 min at 20°C and, once the plasma was obtained, a series of measurements were made. The animals' plasma pools were mixed in the same sodium citrate tube and were then centrifuged and immediately analyzed. Individual plasma samples were aliquoted into two Eppendorf tubes; the sample in the first tube was subjected to an immediate analysis while the one in the other was frozen at −80°C to induce formation of a *superpool*.

### Hematological and Coagulation Tests

FV, PT and APTT determinations were carried out in a STart Max II^®^ viscosity-based coagulometer (Diagnostica Stago S.A.S. Barcelona, Spain) according to the manufacturer's instructions. The programs corresponding to each parameter were strictly followed. To carry out the measurements, a series of cuvettes were placed in the coagulometer (Start 4 Cuve, Diagnostica Stago S.A.S. Barcelona, Spain) to which metal beads were subsequently added (Diagnostica Stago S.A.S. Barcelona, Spain). All reagents were reconstituted according to the manufacturer's instructions. Once all the reagents and the sample were poured into the cuvettes, the reaction was initiated. The determination ended automatically as soon as the clot was formed. At the same time as the determinations were made, positive and negative controls were performed of the System control N/P reagents (Diagnostica Stago S.A.S. Barcelona, Spain).

#### Factor V Determination

A calibration curve was drawn with a commercially available calibrator (Unicalibrator). FV-deficient plasma was used (Sta Deficient V Diagnostica Stago S.A.S. Barcelona, Spain), together with Neoplastine Cl+5 (Diagnostica Stago S.A.S. Barcelona, Spain), with an international sensitivity index (ISI) of 1.27. The metal beads placed in the cuvettes were preheated to 37°C for 3 min. Once reconstituted, Neoplastine Cl+5 was homogenized and preheated to 37°C for 3 min before use. The Owren Koller buffer (Diagnostica Stago S.A.S. Barcelona, Spain) was allowed to stand for 30 min to bring it to room temperature and then perform the corresponding dilutions. A mixture of 50 μL of FV-deficient plasma and 50 μL of the sample to be analyzed (diluted 1:10 in Owren Koller buffer) was introduced in the cuvette. After a 60 s incubation period, 100 μL Neoplastine Cl+5 was added. Calibration curves were calculated using mouse plasma pools following the same procedure as for the commercially available calibrator. Pools were measured using 1:10, 1:20, 1:40, 1:80, and 1:100 dilutions while individual samples were diluted at 1:10 and 1:100. The data obtained was expressed in seconds and percentages.

#### Determination of Prothrombin Time

To calculate the PT, a calibration curve was drawn using a commercially available calibrator (Unicalibrator). Neoplastine Cl+5 with an ISI of 1.27 was used. Metal beads were placed in each well and preheated to 37°C for 3 min. Once reconstituted, Neoplastine Cl+5 was homogenized and preheated at 37°C for 3 min before use. A total of 50 μL of the sample was introduced into the well and, after 60 s incubation, 100 μL Neoplastine Cl+5 was added. The Owren Koller buffer was allowed to stand for 30 min before the relevant dilutions were performed. Calibration curves with mouse plasma pools were created following the same procedure as with the commercially available calibrator. Pools were measured using 1:1, 1:2, 1:3, and 1:4 dilutions; individual samples were diluted at 1:1 and 1:3. The prothrombin time data obtained from individual measurements was expressed in seconds and percentages; the international normalized ratio (INR) was calculated as the ratio between the sample's PT and the reference PT derived from mixing all the individual aliquots frozen at −80°C raised to the ISI power.

#### Determination of the Activated Partial Thromboplastin Time

APTT was measured by placing metal beads in all the cuvettes and preheating them to 37°C for 3 min. A total of 50 μL of a 1:1 dilution of plasma and 50 μL of PPT Automate (Diagnostica Stago S.A.S. Barcelona, Spain) was added to the cuvette; after incubation of the mixture for 180 s, 50 μL of CaCl_2_ 0.025M (Diagnostica Stago S.A.S. Barcelona, Spain) were added. The data obtained was expressed in seconds.

### Statistical Analysis

The statistical analysis was conducted by the Research Support Unit of the IT Department of the Complutense University of Madrid using the SPSS 27, v9.4 statistical package (SAS Institute, Cary, NC, USA). Calibration curves were created using Excel software (Microsoft Office 365) and all graphs were designed with GraphPad Prism 8 software (GraphPad Software, La Jolla, CA, USA). Normal distribution of data was determined using the Kolmogorov-Smirnov test. As regards individual measurements, sex differences were evaluated using Student's *t-*test. Results were considered statistically significant if *p-*value < 0.05.

## Results

### Calibration Studies

**Figure 1:**
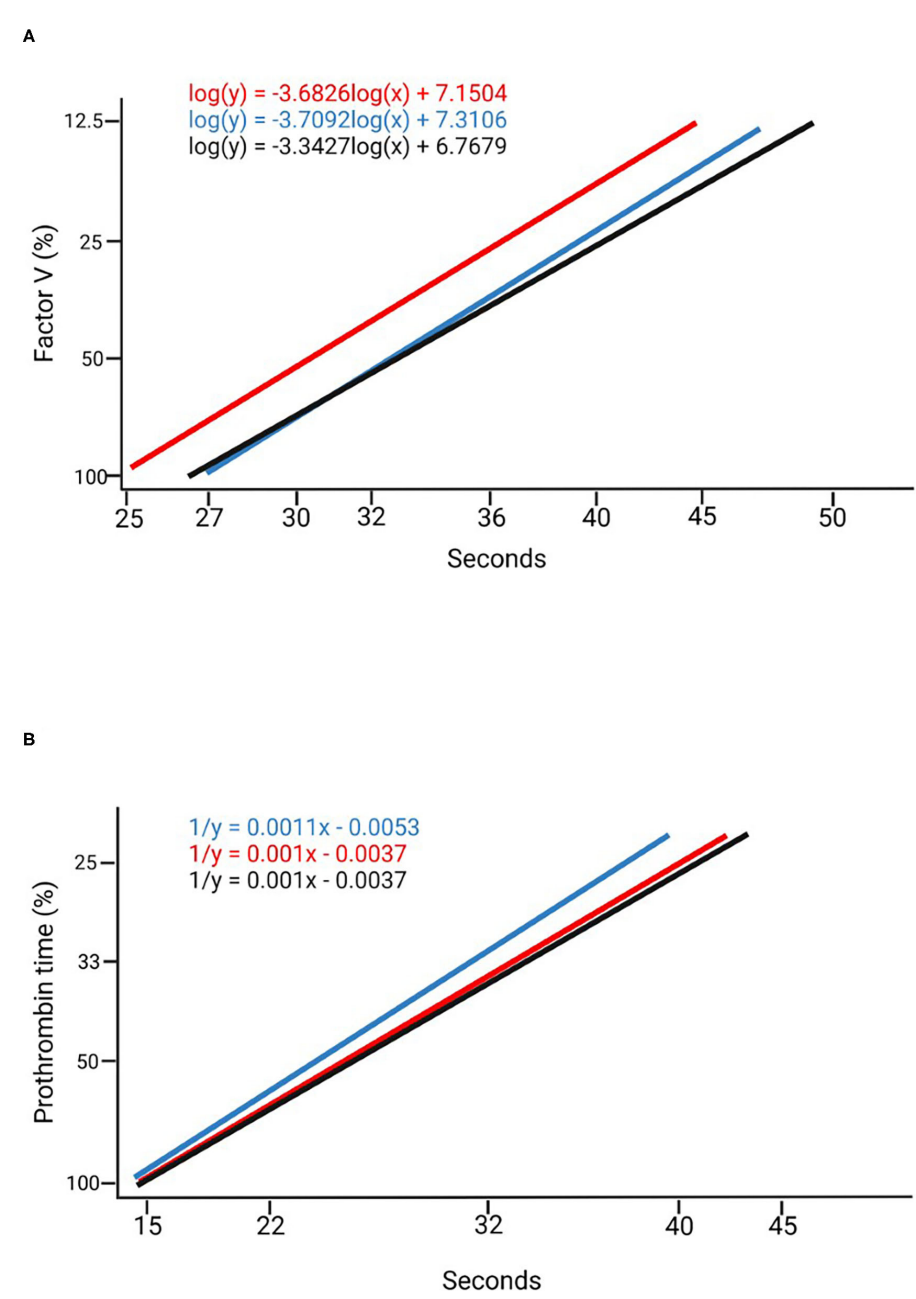
Calibration curves. **(A)** Factor V calibration curves created using a commercially available calibrator (blue), in a 1:100 dilution at Dark 0 (black) and a 1:100 dilution at Ligth 0 (red). **(B)** Prothrombin time calibration curves created with a commercially available calibrator (blue) in a 1:3 dilution at Dark 0 (black) and a 1:3 dilution at Ligth 0 (red).

Using the commercially available calibrator and the plasma pools at different dilution rates (Table 1), measurements were made of six randomly selected individuals (3 males and 3 females) at the beginning of the dark period (dark 0) and at the beginning of the light period (light 0), comparing the values obtained with both methods. Calibration curves were obtained for FV and PT with the commercially available calibrator; the curves were mapped based on the plasma pool determinations at both points in time with the most appropriate dilution (Figure 1). The measurements of the standard calibration curves are presented in Supplementary Table 1.

**Table 1:** Measurements of factor V levels and prothrombin time at the onset of light and at the onset of dark with respect to the commercially available calibrator.

**Factor V**				**Prothrombin Time**			
	**Calibrator**	**Dark 0^a^**	**Ligth 0^b^**		**Calibrator**	**Dark 0**	**Ligth 0**
**Dilution**	**Time (seconds)**			**Dilution**	**Time (seconds)**		
1:10	27.3	13.8^c^	12.3	1:1	27.3	10.0	10.1
1:20		14.1	14.0	1:2		12.9	12.5
1:40		16.3	16.0	1:3		15.6	16.0
1:80		20.0	19.8	1:4		19.0	20.0
1:100		27.3	25.3				

a *Onset of dark*.

b *Onset of light*.

c *A single measurement during the dark period and a single measurement during the light period confirmed the higher clotting rate*.

### Plasma Pool and Individual Sample Studies

**Figure 2:**
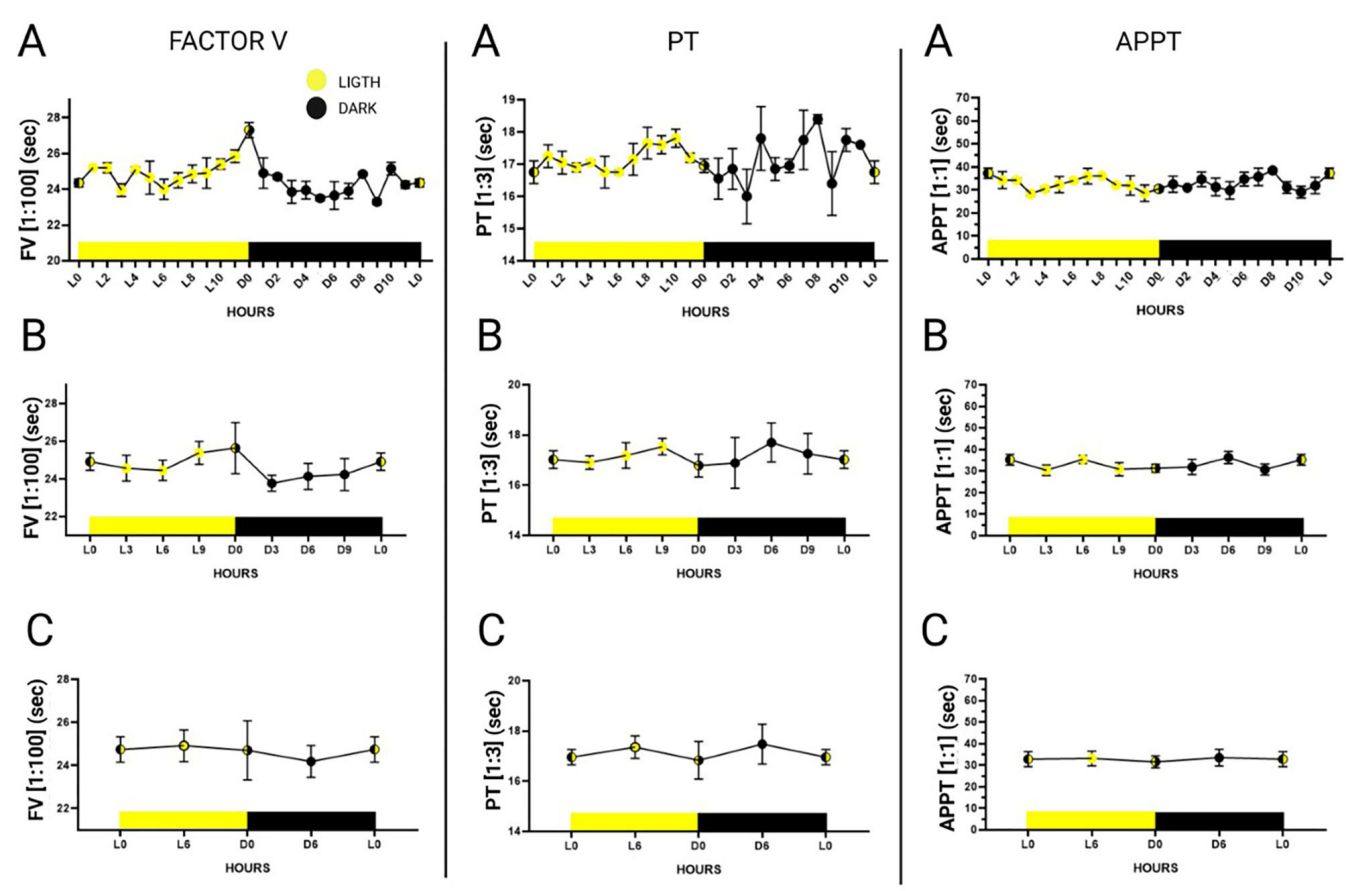
24-h plasma pool-determinations of factor V in a 1:100 dilution, prothrombin time in a 1:3 dilution and activated partial thromboplastin time in a 1:1 dilution. Hours are represented as Dark 0 (onset of dark) to Dark 11 and Ligth 0 (onset of light) to Ligth 11. The graphs were divided according to the statistical groupings analyzed. **(A)** Representation of hourly measurements. **(B)** Combined representation of measurements every 3 h. **(C)** Combined representation of measurements every 6 h. Data is expressed as mean ± standard deviation.

Plasma pool measurements were made every hour during the 24 h of the day (dark 0 to dark 11 and light 0 to light 11), of six randomly selected individuals (3 males and 3 females) using a 1:100 dilution for FV, a 1:3 dilution for PT and a 1:1 dilution for APTT (Figure 2). Six-hour mean values (expressed in seconds) were more stable during the first 6 h of light. Mean FV values during the first 6 h of light were 24.74 s ± 0.58; mean PT values were 16.95 s ± 0.30; and mean APTT values were 32.80 s ± 3.48. The data is represented in Supplementary Table 2.

**Figure 3:**
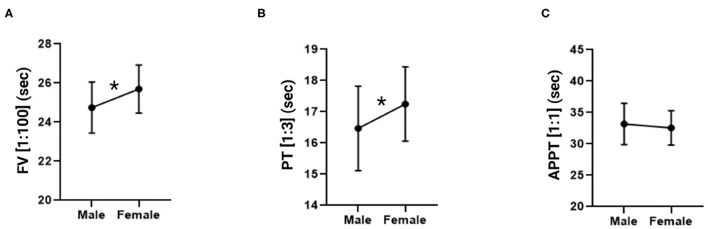
Factor V, prothrombin time and activated partial thromboplastin time measurements in male and female mice. **(A)** Factor V in a 1:100 dilution. **(B)** Prothrombin time in a 1:3 dilution. **(C)** Activated partial thromboplastin time in a 1:1 dilution. The data is expressed as mean ± standard deviation. ^*^p < 0.05 in Student's *t-*test.

An unpaired Student's *t-*test was performed for each of the dependent variables analyzed in individual measurements (FV, PT and APTT) to find out whether there were any differences with respect to sex (male vs. female). Mean FV values (expressed in seconds) were 24.75 ± 1.30 in males and 25.69 ± 1.23 in females. Mean PT values (expressed in seconds) in males and females were 16.47 ± 1.35 and 17.25 ± 1.19, respectively. Finally, as far as APPT is concerned, males exhibited a mean value of 33.18 s ± 3.29 and females a mean value of 32.55 s ± 2.73. The data is shown in Figure 3, indicating the statistical relations and expressing the 1:100 FV, 1:3 PT and 1:1 APTT values in seconds. The data is presented as means ± standard deviation in Table 2. The FV and PT measurements yielded statistically significant differences between the sexes (*p* = 0.0034 and *p* = 0.0157, respectively) while no statistically significant differences were found for the APPT measurements.

**Table 2:** Sex-based measurements of factor V levels, prothrombin time and activated partial thromboplastin time.

**Variable**	**♂**	**♀**	**Δ means^a^**	***p*** **-value^a^**
FV (1:100) (sec)	24.75 ± 1.3^b^	25.69 ± 1.23	0.94	0.0034
PT (1:3) (sec)	16.47 ± 1.35	17.25 ± 1.19	0.78	0.0157
APPT (1:1) (sec)	33.18 ± 3.29	32.55 ± 2.73	0.63	>0.05

a *Difference between the means and the p-value obtained at each Student's t-test*.

b *Data expressed as mean ± SD (n = 33 ♀; n = 33 ♂) (p < 0.05)*.

**Figure 4:**
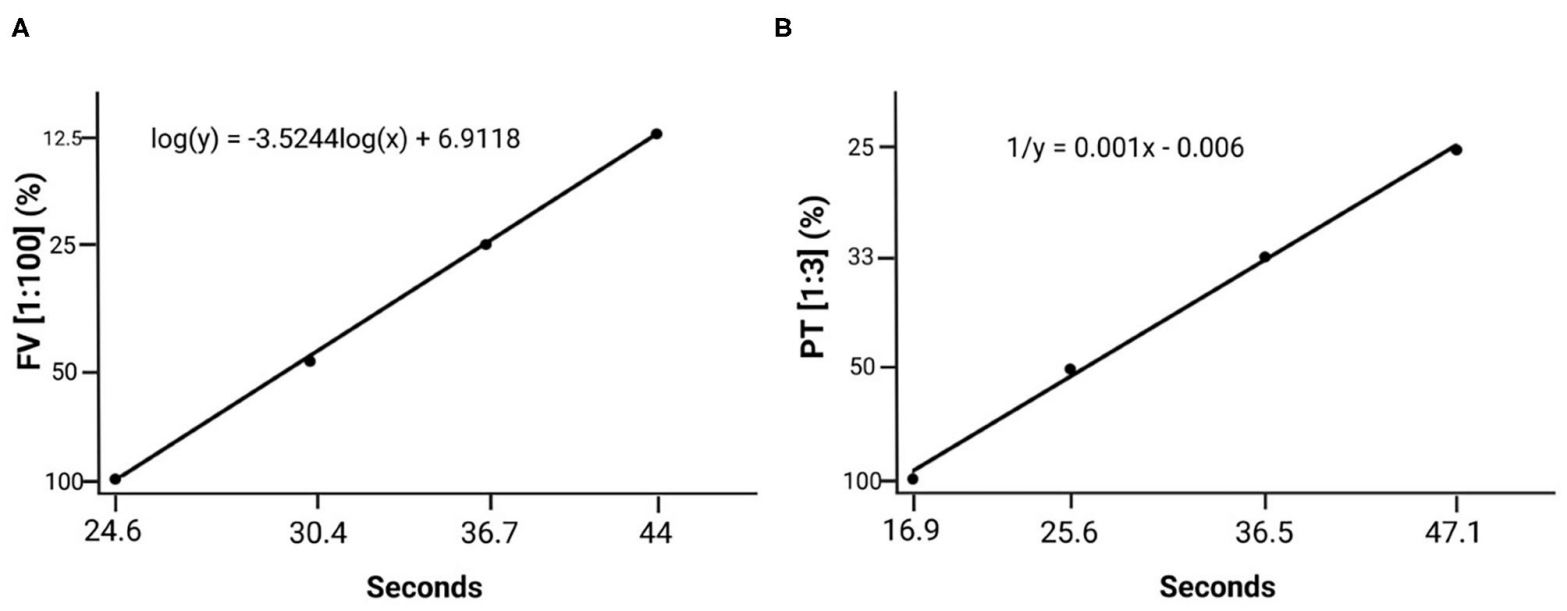
Standard calibration curves created based on the plasma *superpool* prepared with plasma from all the individuals. **(A)** Standard factor V curve (1:100 dilution). **(B)** Standard prothrombin time curve (1:3 dilution).

Calibration curves were drawn applying 1:100 dilutions for FV and 1:3 dilutions for PT on the basis of a *superpool* (Figure 4).

An analysis was made of the statistical values for all 66 individuals in the sample as the differences found between the sexes did not prove clinically significant (Table 3). Mean FV values using the 1:10 dilution were 1,077,260% ± 177.77 (13.65 s ± 0.59), which is 10 times higher than the reference interval established by the manufacturer. Using the 1:100 dilution, a mean FV value of 95.80% ± 18.14 (25.21 s ± 1.34) was obtained. Mean PT values at a 1:1 dilution were 124.10% ± 9.68 (11.29 s ± 0.50) and at a 1:3 dilution they were 104.31% ± 14.52 (16.85 s ± 1.32), both values comprised within the reference interval established by the manufacturers of the diagnostic reagents used. The 1:1 and 1:3 dilution PT data was expressed in terms of the international normalized ratio (INR), taking the measurements of the *superpool* prepared with plasma from all the individuals as the reference plasma measurement in each case. A measurement of 11.2 s was used as plasma reference value for the 1:1 dilution and a measurement of 16.9 s was used for the 1:3 dilution. APPT values were 32.86 s ± 3.01. The data from all the individuals is shown in Supplementary Table 3.

**Table 3:** Measurements*^a^* of factor V, prothrombin time, International Normalized Ratio (INR) and activated partial thromboplastin time.

**Variable**	**Minimum value**	**Maximum value**	**Mean**	**Standard deviation**	**Variation coefficient (%)**	**Median**	**Reference value**
% FV [1:10]	735.0	1,485.00	1,077.260	177.77	16.50	1,049.000	
FV [1:10] (sec)	12.5	15.10	13.650	0.59	4.38	13.700	70–120% (21)^b^ 81–160% (22, 23)^c^
% FV [1:100]	64.0	144.00	95.800	18.14	18.93	92.500	4,632 ± 1,066 mU/mL (14)^d^ 292 ± 8.9 mU/mL (15)^d^
FV [1:100] (sec)	22.3	28.10	25.210	1.34	5.32	25.300	
% PT [1:1]	105.0	145.00	124.100	9.68	7.80	124.500	
PT [1:1] (sec)	10.3	12.50	11.290	0.50	4.50	11.250	>70% (24)^b^
% PT [1:3]	74.0	143.00	104.310	14.52	13.92	101.500	10-14 s (17, 22)^c^
PT [1:3] (sec)	14.1	20.60	16.850	1.32	7.85	16.950	10.19 s ± 0.48 (19)^d^
INR [1:1]	0.9	1.15	1.0160	0.06	5.72	1.011	No data^b^ 0.8–1.2 (25)^c^ No data^d^
INR [1:3]	0.8	1.28	0.996	0.10	9.98	1.002	
APPT [1:1] (sec)	27.0	41.20	32.860	3.01	9.17	32.400	32.7 s ± 2.5^b^ 30-33 s (22)^c^ 20–>30 s (19)^d^

a *All data followed a normal distribution (n = 66)*.

b *Recommended by the reagent manufacturer*.

c *Referenced for humans*.

d *Referenced for mice*.

## Discussion

This study describes a novel strategy aimed at accurately determining and standardizing the measurement of coagulation FV levels, PT and APTT in *Mus musculus*. A large sample size was used, and the INR value was calculated for the first time in a mouse model. Moreover, a coagulometer routinely used in clinical practice was employed, together with human diagnostic reagents. Although reagents used for coagulometric diagnosis of human samples may be applied to mouse models (26, 27), they have limitations in terms of the sample amounts that may be analyzed (18, 28).

This is the first study to describe a method for determining FV levels in mice based on a large sample size, which is essential to reach statistical significance. Accuracy is essential when determining FV levels in FV-deficient pathological models. Moreover, for plasma transfusions, it is necessary to preserve the individuals' quality of life and minimize the incidence of spontaneous bleeds (29, 30).

Factor V deficiency results in bleeds that may occur spontaneously or as a result of trauma or invasive medical procedures (11, 31–33). The treatment of choice is currently based on infusions of fresh frozen plasma (FFP) (34), or coagulation factor concentrates at precisely tested levels (35). *In vitro* studies have suggested that certain plasma concentrates are able to correct the disease, inducing normalized FV values (36). Human plasma reference concentrations (determined through coagulometric methods) stand at 7 mg/dL (81–160%) (11, 22, 23). FV levels have also been measured by means of thromboelastometry (37).

Unlike other congenital coagulopathies such as hemophilia (38–42), there is no commercially- available recombinant FV concentrate to treat FV deficiency (34, 43). Not many coagulation factor deficiencies have been reported in the field of veterinary medicine. Some coagulopathies have been described in dogs and, to a lesser extent, in cats; hemophilia A and hemophilia B being the most prevalent (44–47). Treatments employed in veterinary medicine to address these conditions consist in infusions of FFP (48) or cryoprecipitates (49, 50).

The measurements of FV levels in this study, made at a 1:10 dilution, yielded very high levels as compared with the levels found by other authors in humans (14–16). The PT and APTT values measured were also high, although these tend to be lineage- and sex-dependent (19). When obtaining such values, calibration curves must be created for each species, as must also be done when studying humans (27). In the case of the very short PTs or APTTs found in some species, which are typically correlated with high clotting rates, a correction must be applied in the dilutions, as suggested by some authors (14, 19). In this way, a curve based on human samples (where clotting times are extrapolated) may be adapted to a mouse protocol with more frequent measurement intervals and lower slopes. In humans, PT values tend to range between 10 and 14 seconds, APPT values between 35 and 42 seconds (17, 22) and FV values between 81–160% of coagulometric activity with respect to control plasma (22, 23).

Calibration curves were drawn for two points in time, one of them was the onset of the light period (Ligth 0) and the other the onset of the dark period (Dark 0). Comparisons were made to select the curve that was closer to the calibration curve for humans. Minimally serialized measurements provide invaluable information about the time at which blood extractions should be performed. Due to the nocturnal lifestyle of *Mus musculus* (51), the greatest fluctuations between the measurements were observed during the night and the most stable values during the day. Some authors (52) have reported alterations in the levels of some coagulation factors depending on the duration of the light period; such alterations varying across different individuals, with signs of hypercoagulability observed during the evening. Some studies, however, have shown that differences are very much lineage-dependent and, for parameters such as PT or APPT, they are not statistically significant with respect to circadian rhythm (53). Our results could help clarify this controversy as the mentioned studies carried out their measurements every 3–6 h while our results, based on hourly measurements, can be considered truer to reality. Indeed, our method allowed detection of changes that would have gone unnoticed if less frequent measurements had been made.

This study also found differences between the values observed at 1 h and those recorded at the next hour, providing a clear picture of the evolution of the different parameters during the day and making it possible to determine the most stable (and least unpredictable) times of day to extract blood samples from each individual. In addition, this allows adapting the murine to the human circadian cycle (51), establishing the first 6 h of light as the most stable period.

Application of Student's *t-*tests to the FV and PT measurements, using the 1:100 and 1:3 dilutions respectively, found statistically significant sex-dependent differences, with clotting rates presenting with higher values in males than in females. Values were in both cases within the physiological range. These results are in line with those reported by Peters et al. (19). Moreover, sex-dependent differences were also described in human medicine for some coagulometric parameters as a function of the drugs patients were on (54). In any case, these sex-related differences were not statistically significant as the variations observed were very small (e.g., clotting time variations were of one second). No differences were found for APTT as the range for mice stands between 20 and more than 30 seconds, which represents a greater dispersion than could be explained by a greater deviation of this parameter and its non-correlation with the sex variable (18).

Moreover, an analysis of the plasma pools corresponding to the first hours of light showed that the individual measurements obtained for all the parameters studied fell within the reference intervals. The parameters were analyzed using both the dilution recommended by the manufacturer for human samples and the dilutions tested as part of the present mouse protocol in order to compare the results of the mouse model with those obtained from human samples (14, 19) and to obtain the measurements for our mouse protocol. In line with the procedure used for human diagnostic parameters, PT measurements must be expressed as INR (0.8–1.2) (55). Our study quantifies for the first time the INR, both at 1:1 and at 1:3 dilutions, with values falling within reference intervals. Although regularly employed in clinical practice with anticoagulated patients (56), use the INR in mice protocols has never been described, nor has its diagnostic value been clearly established. This first-ever inclusion of INR in a murine model responds to our belief that using the INR could contribute to standardizing the measurement of the parameters discussed in the study.

## Conclusions

The standardization of the measurement of FV, PT and APPT presented in this study could be useful in animal models such as FV deficiency pathological animal model for the accurate determination of FV levels.

The need to standardize measurement methods stems from the fact that FV levels in mice exhibit markedly increased values as compared with those in humans. Moreover, human-specific diagnostic tools such as human-specific reagents and coagulometers were adapted in our study to murine protocols and the INR was used as a standardization tool for the mouse model.

In addition, following standard human medicine practice, and contrary to previous reports in the literature, seconds and percentages were established as mouse FV level measurement units.

The strategy put forward in this article can help establish reference intervals and detect small differences between healthy and diseased individuals in the context of veterinary or human biomedicine, in transfusional medicine, or in the pathological models used for congenital coagulopathies.

## Data Availability Statement

The original contributions presented in the study are included in the article/Supplementary Material, further inquiries can be directed to the corresponding author.

## Ethics Statement

The animal study was reviewed and approved by Royal Decree 53/2013 and Directive 2010/63/EU. All procedures used were approved by the School of Veterinary Medicine of the Complutense University of Madrid, the Ethics Committee on Animal Experimentation of the Complutense University, and the Madrid Regional Government (PROEX 258.1/21).

## Author Contributions

JADPM contributed to the design of the experiments and participated in the collection, analysis, and interpretation of the data. He also contributed to drafting the manuscript and preparing the figures and tables. AL and LR contributed to the conception and design of the experiments, participated in the data analysis, contributed the reagents, materials and analytical tools, collaborated in drafting the manuscript and preparing the figures and tables, and reviewed successive drafts of the paper. AL is the principal investigator of the project and obtained the funds required for the project. All authors have read and agreed to the published version of the manuscript.

## Funding

This study was supported by ASDEFAV, the Association for Research and Cure of Factor V deficiency. The manuscript was prepared within the framework of a predoctoral research internship contract (CT63/19-CT64/19), awarded by the Complutense University of Madrid and Banco Santander.

## Conflict of Interest

The authors declare that the research was conducted in the absence of any commercial or financial relationships that could be construed as a potential conflict of interest.

## Publisher's Note

All claims expressed in this article are solely those of the authors and do not necessarily represent those of their affiliated organizations, or those of the publisher, the editors and the reviewers. Any product that may be evaluated in this article, or claim that may be made by its manufacturer, is not guaranteed or endorsed by the publisher.
