# Supplementary material for: Standardization of Coagulation Factor V Reference Intervals, Prothrombin Time, and Activated Partial Thromboplastin Time in Mice for Use in Factor V Deficiency Pathological Models
Source: Front Vet Sci. 2022 Mar 28;9:846216. doi: 10.3389/fvets.2022.846216 (PMC8995772; doi:10.3389/fvets.2022.846216)
Supplement: Supplementary file 1 [file Data_Sheet_1.docx]

**Supplementary Table 1 |** Standard calibration curves relative to Figure 1.

Standard factor V and prothrombin time (sec) curves at the onset of dark and at the onset of light mapped

on the basis of the commercially available calibrator.

| **Factor V** | | | | **Prothrombin time** | | | |
| --- | --- | --- | --- | --- | --- | --- | --- |
|  |  |  |  |  |  |  |  |
| **%FV** | **Calibrator** | **Dark 0^a^ [1:100]** | **Ligth 0^b^ [1:100]** | **%PT** | **Calibrator** | **Dark 0 [1:3]** | **Ligth 0 [1:3]** |
| 100 | 27.3 | 27.3^c^ | 25.3 | 100 | 14.5 | 16.2 | 16.2 |
| 50 | 31.9 | 32.2 | 29.9 | 50 | 21.6 | 22.0 | 21.9 |
| 25 | 39.9 | 39.6 | 36.2 | 33 | 31.6 | 33.4 | 31.7 |
| 12.5 | 47.1 | 50.6 | 44.4 | 25 | 40.9 | 45.7 | 45.3 |

^a^Onset of dark.

^b^Onset of light.

^c^One single measurement (sec) during the dark period and one single measurement during the light period confirm the calibration curve with

the selected dilution.

**Supplementary Table 2 |** Determination of the dilutions selected for factor V, prothrombin

time and activated partial thromboplastin time over 24 hours (expressed in seconds) based on

plasma pools from 6 randomly selected individuals (3 males and 3 females).

| **Hours^a^** | **Factor V [1:100] (sec)** | **PT [1:3] (sec)** | **APPT [1:1] (sec)** |
| --- | --- | --- | --- |
|  |  |  |  |
| **L0** | 24.2 | 16.5 | 35.7 |
| **L0** | 24.5 | 17.0 | 38.8 |
| **L1** | 25.2 | 17.0 | 31.6 |
| **L1** | 25.2 | 17.5 | 36.8 |
| **L2** | 25.4 | 17.3 | 34.9 |
| **L2** | 25.0 | 16.8 | 33.7 |
| **L3** | 23.7 | 17.0 | 27.7 |
| **L3** | 24.2 | 16.8 | 28.9 |
| **L4** | 25.2 | 17.0 | 29.8 |
| **L4** | 25.0 | 17.1 | 31.1 |
| **L5** | 24.0 | 17.1 | 29.8 |
| **L5** | 25.3 | 16.4 | 34.8 |
| **L6** | 24.4 | 16.7 | 33.6 |
| **L6** | 23.6 | 16.8 | 34.6 |
| **L7** | 24.8 | 17.5 | 33.6 |
| **L7** | 24.2 | 16.8 | 38.4 |
| **L8** | 25.2 | 18.0 | 36.2 |
| **L8** | 24.5 | 17.3 | 36.1 |
| **L9** | 25.5 | 17.4 | 32.0 |
| **L9** | 24.3 | 17.8 | 32.2 |
| **L10** | 25.2 | 17.6 | 28.9 |
| **L10** | 25.6 | 18.0 | 34.9 |
| **L11** | 26.1 | 17.3 | 26.0 |
| **L11** | 25.6 | 17.1 | 31.1 |
| **D0** | 27.6 | 17.1 | 29.7 |
| **D0** | 27.0 | 16.8 | 31.3 |
| **D1** | 25.5 | 17.0 | 35.0 |
| **D1** | 24.3 | 16.1 | 30.0 |
| **D2** | 24.8 | 17.3 | 30.8 |
| **D2** | 24.6 | 16.4 | 31.1 |
| **D3** | 24.3 | 16.6 | 36.8 |
| **D3** | 23.4 | 15.4 | 32.4 |
| **D4** | 23.6 | 18.5 | 28.5 |
| **D4** | 24.3 | 17.1 | 34.0 |
| **D5** | 23.6 | 16.6 | 32.5 |
| **D5** | 23.4 | 17.1 | 27.1 |
| **D6** | 23.1 | 17.1 | 32.6 |
| **D6** | 24.2 | 16.8 | 36.8 |
| **D7** | 23.6 | 17.1 | 33.0 |
| **D7** | 24.2 | 18.4 | 38.4 |
| **D8** | 24.8 | 18.3 | 39.1 |
| **D8** | 24.9 | 18.5 | 37.8 |
| **D9** | 23.2 | 15.7 | 29.3 |
| **D9** | 23.4 | 17.1 | 32.9 |
| **D10** | 24.9 | 17.5 | 27.3 |
| **D10** | 25.4 | 18.0 | 30.9 |
| **D11** | 24.4 | 17.6 | 29.4 |
| **D11** | 24.1 | 17.6 | 34.4 |

^a^Measurements were made over a 12-hour light (L) period and over a 12-hour dark (D) period.

**Supplementary Table 3 |** Factor V measurements (expressed as % and seconds), prothrombin time measurements (expressed as %, seconds and INR) and activated partial thromboplastin time measurements (expressed in seconds) for the whole sample (66 individuals).

| **Sex** | **Weight^a^** | **FV [1:10] (%)^b^** | **FV [1:10] (sec)** | **FV [1:100] (%)^c^** | **FV [1:100] (sec)** | **PT [1:1] (%)^b^** | **PT [1:1] (sec)** | **PT [1:3] (%)^c^** | **PT [1:3] (sec)** | **INR^d^ [1:1]** | **INR^d^ [1:3]** | **APPT [1:1] (sec)** |
| --- | --- | --- | --- | --- | --- | --- | --- | --- | --- | --- | --- | --- |
|  | 30.8 | 1,146 | 13.4 | 102 | 24.6 | 122 | 11.4 | 123 | 15.2 | 1.029 | 0.873 | 34.9 |
|  | 30.0 | 994 | 13.9 | 83 | 26.1 | 105 | 12.5 | 112 | 16.0 | 1.156 | 0.932 | 36.8 |
|  | 39.2 | 1,176 | 13.3 | 108 | 24.2 | 126 | 11.2 | 123 | 15.2 | 1.006 | 0.873 | 32.4 |
|  | 38.8 | 891 | 14.3 | 101 | 24.7 | 125 | 11.2 | 95 | 17.6 | 1.006 | 1.052 | 34.1 |
|  | 36.0 | 1,057 | 13.7 | 105 | 24.4 | 115 | 11.8 | 112 | 16.0 | 1.075 | 0.932 | 32.0 |
|  | 41.2 | 912 | 14.2 | 90 | 25.5 | 129 | 11.0 | 110 | 16.2 | 0.983 | 0.947 | 37.6 |
|  | 32.0 | 1,271 | 13.0 | 117 | 23.7 | 119 | 11.5 | 112 | 16.0 | 1.040 | 0.932 | 29.9 |
|  | 40.0 | 1,101 | 13.5 | 97 | 25.0 | 108 | 12.3 | 97 | 17.4 | 1.133 | 1.036 | 35.6 |
|  | 41.0 | 1,196 | 13.2 | 117 | 23.7 | 138 | 10.6 | 101 | 17.0 | 0.938 | 1.006 | 31.2 |
|  | 38.7 | 1,060 | 13.7 | 97 | 25.0 | 128 | 11.1 | 115 | 15.8 | 0.994 | 0.917 | 32.4 |
|  | 31.4 | 1,228 | 13.1 | 118 | 23.6 | 143 | 10.4 | 114 | 15.9 | 0.915 | 0.924 | 33.4 |
|  | 31.4 | 882 | 14.3 | 74 | 27.0 | 115 | 11.8 | 83 | 19.2 | 1.075 | 1.174 | 33.9 |
|  | 36.6 | 1,104 | 13.5 | 90 | 25.5 | 131 | 10.9 | 105 | 16.6 | 0.972 | 0.976 | 31.3 |
| **♀** | 32.1 | 1,141 | 13.4 | 91 | 25.4 | 133 | 10.8 | 93 | 17.9 | 0.960 | 1.074 | 31.3 |
|  | 30.7 | 900 | 14.3 | 83 | 26.1 | 110 | 12.1 | 97 | 17.4 | 1.109 | 1.036 | 34.1 |
|  | 30.5 | 916 | 14.2 | 83 | 26.1 | 108 | 12.2 | 97 | 17.4 | 1.121 | 1.036 | 34.6 |
|  | 30.9 | 1,151 | 13.3 | 89 | 25.6 | 138 | 10.6 | 98 | 17.3 | 0.938 | 1.029 | 29.5 |
|  | 46.2 | 1,074 | 13.6 | 83 | 26.1 | 141 | 10.5 | 94 | 17.7 | 0.927 | 1.059 | 30.4 |
|  | 30.2 | 1,040 | 13.7 | 90 | 25.5 | 125 | 11.2 | 101 | 17.0 | 1.006 | 1.006 | 31.3 |
|  | 38.3 | 1,039 | 13.7 | 88 | 25.7 | 131 | 10.9 | 97 | 17.4 | 0.972 | 1.036 | 30.3 |
|  | 26.6 | 830 | 14.5 | 67 | 27.7 | 115 | 11.8 | 83 | 19.2 | 1.075 | 1.174 | 33.9 |
|  | 34.6 | 806 | 14.7 | 67 | 27.7 | 110 | 12.1 | 74 | 20.6 | 1.109 | 1.284 | 35.3 |
|  | 33.0 | 790 | 14.8 | 70 | 27.4 | 112 | 11.9 | 97 | 17.4 | 1.086 | 1.036 | 37.4 |
|  | 43.0 | 832 | 14.6 | 77 | 26.7 | 116 | 11.7 | 94 | 17.7 | 1.063 | 1.059 | 37.7 |
|  | 54.3 | 1,032 | 13.8 | 101 | 24.7 | 126 | 11.2 | 100 | 17.1 | 1.006 | 1.014 | 31.3 |
|  | 43.5 | 1,274 | 13.0 | 102 | 24.6 | 128 | 11.1 | 99 | 17.2 | 0.994 | 1.021 | 28.1 |
|  | 35.1 | 837 | 14.6 | 81 | 26.3 | 117 | 11.7 | 106 | 16.5 | 1.063 | 0.969 | 32.9 |
|  | 29.0 | 1,025 | 13.8 | 81 | 26.3 | 109 | 12.2 | 86 | 18.7 | 1.121 | 1.136 | 31.2 |
|  | 28.6 | 953 | 14.1 | 67 | 27.7 | 125 | 11.2 | 99 | 17.2 | 1.006 | 1.021 | 29.8 |
|  | 27.5 | 831 | 14.6 | 64 | 28.1 | 117 | 11.6 | 85 | 18.8 | 1.052 | 1.143 | 33.0 |
|  | 24.8 | 735 | 15.1 | 99 | 24.8 | 129 | 11.0 | 88 | 18.5 | 0.983 | 1.120 | 30.0 |
|  | 24.9 | 1,154 | 13.3 | 86 | 25.8 | 144 | 10.4 | 102 | 16.9 | 0.915 | 0.999 | 27.1 |
|  | 24.8 | 984 | 13.9 | 78 | 26.6 | 121 | 11.4 | 99 | 17.2 | 1.029 | 1.021 | 29.5 |
|  | 45.0 | 1,004 | 13.9 | 76 | 26.8 | 119 | 11.5 | 110 | 16.2 | 1.040 | 0.947 | 38.0 |
|  | 49.0 | 1,009 | 13.8 | 76 | 26.8 | 121 | 11.4 | 110 | 16.2 | 1.029 | 0.947 | 40.3 |
|  | 46.0 | 1,102 | 13.5 | 94 | 25.2 | 127 | 11.1 | 116 | 15.7 | 0.994 | 0.910 | 32.0 |
|  | 51.2 | 984 | 13.9 | 89 | 25.6 | 124 | 11.3 | 111 | 16.1 | 1.017 | 0.939 | 27.0 |
|  | 47.0 | 1,257 | 13.0 | 112 | 24.0 | 131 | 10.9 | 137 | 14.4 | 0.972 | 0.815 | 29.6 |
|  | 47.5 | 1,457 | 12.5 | 126 | 23.2 | 130 | 11.0 | 137 | 14.4 | 0.983 | 0.815 | 41.2 |
|  | 43.0 | 1,362 | 12.8 | 138 | 22.6 | 126 | 11.2 | 137 | 14.4 | 1.006 | 0.815 | 30.9 |
|  | 45.5 | 1,252 | 13.1 | 117 | 23.7 | 133 | 10.8 | 119 | 15.5 | 0.960 | 0.895 | 29.1 |
|  | 44.5 | 1,485 | 12.5 | 144 | 22.3 | 145 | 10.3 | 125 | 15.1 | 0.904 | 0.866 | 28.5 |
|  | 42.0 | 1,480 | 12.5 | 138 | 22.6 | 129 | 11.0 | 143 | 14.1 | 0.983 | 0.794 | 30.9 |
|  | 45.5 | 1,341 | 12.8 | 117 | 23.7 | 123 | 11.3 | 132 | 14.7 | 1.017 | 0.837 | 33.6 |
|  | 50.2 | 925 | 14.2 | 88 | 25.7 | 117 | 11.6 | 106 | 16.5 | 1.052 | 0.969 | 36.1 |
|  | 35.2 | 957 | 14.0 | 86 | 25.8 | 114 | 11.8 | 101 | 17.0 | 1.075 | 1.006 | 37.4 |
|  | 34.0 | 1,077 | 13.6 | 94 | 25.2 | 120 | 11.5 | 111 | 16.1 | 1.040 | 0.939 | 33.9 |
|  | 44.9 | 953 | 14.1 | 86 | 25.8 | 116 | 11.7 | 104 | 16.7 | 1.063 | 0.984 | 36.7 |
| **♂** | 38.1 | 990 | 13.9 | 94 | 25.2 | 116 | 11.7 | 106 | 16.5 | 1.063 | 0.969 | 36.0 |
|  | 51.6 | 935 | 14.1 | 90 | 25.5 | 117 | 11.6 | 94 | 17.7 | 1.052 | 1.059 | 36.6 |
|  | 53.2 | 1,004 | 13.9 | 95 | 25.1 | 128 | 11.1 | 106 | 16.5 | 0.994 | 0.969 | 34.3 |
|  | 49.3 | 1,077 | 13.6 | 113 | 23.9 | 124 | 11.3 | 95 | 17.6 | 1.017 | 1.052 | 35.2 |
|  | 44.4 | 1,180 | 13.3 | 115 | 23.8 | 121 | 11.4 | 100 | 17.1 | 1.029 | 1.014 | 35.0 |
|  | 53.5 | 943 | 14.1 | 105 | 24.4 | 137 | 10.6 | 110 | 16.2 | 0.938 | 0.947 | 33.6 |
|  | 35.0 | 907 | 14.2 | 64 | 28.1 | 116 | 11.7 | 103 | 16.8 | 1.063 | 0.991 | 31.9 |
|  | 40.5 | 1,317 | 12.9 | 117 | 23.7 | 133 | 10.8 | 92 | 18.0 | 0.960 | 1.082 | 32.5 |
|  | 46.2 | 1,041 | 13.7 | 88 | 25.7 | 117 | 11.6 | 93 | 17.9 | 1.052 | 1.074 | 31.0 |
|  | 46.5 | 1,387 | 12.7 | 126 | 23.2 | 135 | 10.7 | 128 | 14.9 | 0.949 | 0.851 | 32.4 |
|  | 36.1 | 1,223 | 13.1 | 97 | 25.0 | 126 | 11.1 | 78 | 20.0 | 0.994 | 1.237 | 32.6 |
|  | 40.2 | 1,109 | 13.5 | 91 | 25.4 | 114 | 11.9 | 96 | 17.5 | 1.086 | 1.044 | 31.9 |
|  | 38.1 | 1,444 | 12.6 | 108 | 24.2 | 140 | 10.5 | 109 | 16.3 | 0.927 | 0.954 | 31.8 |
|  | 39.5 | 1,197 | 13.2 | 110 | 24.1 | 142 | 10.5 | 103 | 16.8 | 0.927 | 0.991 | 31.0 |
|  | 44.4 | 1,023 | 13.8 | 91 | 25.4 | 121 | 11.4 | 85 | 18.8 | 1.029 | 1.143 | 30.4 |
|  | 45.3 | 1,036 | 13.7 | 99 | 24.8 | 116 | 11.7 | 91 | 18.1 | 1.063 | 1.090 | 32.5 |
|  | 45.0 | 1,060 | 13.7 | 86 | 25.8 | 126 | 11.1 | 97 | 17.4 | 0.994 | 1.036 | 31.1 |
|  | 44.5 | 1,219 | 13.2 | 107 | 24.3 | 128 | 11.1 | 109 | 16.3 | 0.994 | 0.954 | 30 |

^a^Weight in grams at the time of individual extraction.

^b^Percentage obtained from the standard curve based on commercially available calibrators used for human diagnosis.

^c^Percentage obtained from the standard curve based on the plasma *superpool* at the selected dilutions.

^d^INR calculated as the ratio between the PT value obtained from the sample and the reference PT obtained from the mixture of all the individual aliquots, raised to the ISI value.
